# Evaluation of Keypoint Descriptors for Flight Simulator Cockpit Elements: WrightBroS Database

**DOI:** 10.3390/s21227687

**Published:** 2021-11-19

**Authors:** Karolina Nurzynska, Przemysław Skurowski, Magdalena Pawlyta, Krzysztof Cyran

**Affiliations:** Faculty of Automatic Control, Electronics and Computer Science, Silesian University of Technology, 44-100 Gliwice, Poland; Przemyslaw.Skurowski@polsl.pl (P.S.); Magdalena.Pawlyta@polsl.pl (M.P.); Krzysztof.Cyran@polsl.pl (K.C.)

**Keywords:** keypoint descriptors, local feature classification, cockpit devices database, feature vectors matching

## Abstract

The goal of the WrightBroS project is to design a system supporting the training of pilots in a flight simulator. The desired software should work on smart glasses supplementing the visual information with augmented reality data, displaying, for instance, additional training information or descriptions of visible devices in real time. Therefore, the rapid recognition of observed objects and their exact positioning is crucial for successful deployment. The keypoint descriptor approach is a natural framework that is used for this purpose. For this to be applied, the thorough examination of specific keypoint location methods and types of keypoint descriptors is required first, as these are essential factors that affect the overall accuracy of the approach. In the presented research, we prepared a dedicated database presenting 27 various devices of flight simulator. Then, we used it to compare existing state-of-the-art techniques and verify their applicability. We investigated the time necessary for the computation of a keypoint position, the time needed for the preparation of a descriptor, and the classification accuracy of the considered approaches. In total, we compared the outcomes of 12 keypoint location methods and 10 keypoint descriptors. The best scores recorded for our database were almost 96% for a combination of the ORB method for keypoint localization followed by the BRISK approach as a descriptor.

## 1. Introduction

This manuscript describes research aiming at finding a rapid and efficient method for the classification of flight simulator elements in the cockpit. This is part of a project that aims to design an augmented reality (AR) system for the training of pilots and the efficient maintenance of the flight simulator. The AR will be presented in parallel with the cockpit interior to the user. In addition to simply displaying the static information to familiarize the user with the available flight procedures, the system will interactively support the pilot in task execution training and testing. In interactive mode, the system will assist technicians in performing procedures that are needed for simulator maintenance. The described software will work based on mixed reality smart glasses—e.g., HoloLens and Oculus. As a consequence, the chosen algorithms must work on the CPU. This is because when a device is equipped with a GPU, it is not accessible for external use.

With these project goals in mind, we evaluated the latest state-of-the-art methods used for image description. In our initial literature overview, we found that using keypoint descriptors should be a beneficial approach. However, the data characteristics should verify their applicability [1,2,3,4]. Considering the huge number of solutions that allow objects to be compared in images, we decided to investigate the differences that influence the effectiveness of image description in the presented problem. Attempts to find an answer to this question in the literature turned out to be futile because most comparative studies limit themselves to the use of a few methods, and it is difficult to draw unequivocal conclusions. This situation is worsened by the fact that many of these studies use different datasets, making the results difficult to compare. Additionally, the presented conclusions were sometimes contradictory, which suggests that the performance of the method depends on the analyzed data. Therefore, it is necessary to carry out research on the use of various methods on one data set to enable their in-depth comparison.

In traditional approaches to object recognition, two steps are used to build the feature vector. In the first step, these methods determine a set of characteristic locations that describe the object well. Next, for each location the keypoint descriptor is calculated. As a consequence, a set of feature vectors is used to describe each object, but only a few of these are sufficient for recognition. This surplus of descriptors is necessary, as, in the case of object rotation or partial occlusion, not all features are available. Recognition is achieved using k-nearest neighbours.

Finding an exact location of an object within a larger scene is a very important problem in the field of computer vision. Therefore, many attempts to develop an optimal solution have been made. The nature of region sampling differs between approaches. However, all of them try to find regions characterized by rapid changes in illumination that correspond to edges, corners, or isolated points. Moreover, these candidate points should be clearly visible at various scales. Candidates found close to each other are also removed. The keypoint descriptor gathers the information from the pixel distribution around the location. It may use the information derived from the gradient on the image patch, store the information about the permutation of pixel values, or derive a statistical distribution for these data.

In this study, the most representative keypoint location and description methods were chosen, implemented, and tested, and we report our results and conclusions here. In addition, to make our work well suited to the task, we prepared a dedicated database. This database consists of thousands of images of 27 selected devices installed in the cockpit of a flight simulator. Each element was photographed under varying lighting conditions, with different camera parameters, using various acquisition qualities. The scenarios used for the acquisition were chosen to reflect situations that might be encountered in everyday life; therefore., they include rotation, translation, and other types of movement.

The contributions of this work are the following:

1 The preparation of a novel database describing the flight simulator cockpit elements;
2 The evaluation of the time and repeatability of the keypoint location methods most widely used in the literature;
3 The proposal to calculat e keypoint descriptors for the best methods of locating keypoint instead of those originally proposed, which, to the best of our knowledge, have not previously been considered;
4 The evaluation of the keypoint description accuracy depending on the method used for finding the localization.

We start this article with a detailed description of the method evaluated during the experiments in Section 2. This section also introduces the WrightBroS database and gives basic information about the Oxford University Database. Next, Section 3 presents the results of the experiments conducted, supplemented by a thorough discussion. Finally, Section 4 draws the conclusions.

## 2. Material and Methods

This section describes the theoretical basis of the methods considered for flight simulator device description. It introduces the WrightBroS database and presents a baseline database that has been in several experiments relating to image description, the Oxford University Database. In this section, we also present the methodology used for the classification of images.

### 2.1. Object Recognition with Keypoint Description

Object classification is based on discriminating between features that describe objects. These features should include the most characteristic features of the item but should also include more general features for objects of its type. Moreover, the algorithm should work correctly in the event of the occlusion or invisibility of some parts of the object. Over several decades, many solutions based on keypoint descriptors have been introduced to address this problem [5,6,7,8,9].

First, these methods were dedicated to achieving simple object detection. Later, the range of possible application s of these algorithms grew significantly. There exist examples of text detection in complex backgrounds [10] as well as methods designed for face recognition [11,12]. Other solutions enable texture recognition [13] or allow for the better matching of remotely sensed images [14]. Recently, research has moved in the direction of precise texture localization [15]. It is also worth mentioning that, according to the conclusions of [16,17], hand-crafted methods tend to outperform deep neural network-based methods.

#### 2.1.1. Keypoint Location

##### Derivative Approach

One of the earliest method s for keypoint location was the Harris corner detector [18], which determines the average changes in image intensities within a moving window, *w*, applied to the image *I*. The following equation describes the energy *E* of the keypoint:

(1) $$E\left(x,y\right)=\sum_{u,v}w\left(u,v\right){\left|I\left(x+u,y+v\right)-I\left(x,y\right)\right|}^{2},$$

where only four shifts are considered: (u,v)=(1,0),(1,1),(0,1),(−1,1). This can be improved by first introducing derivative calculations, such as applying a Sobel filter to the image spot and reformulating the equation as follows:

(2) $$E\left(x,y\right)=Ax^{2}+2Cxy+By^{2},$$

where *A* is a horizontal, *B* a vertical, and *C* a diagonal derivative. In order to diminish the influence of noise on the computation, the corner response can be formulated as:

(3) $$E\left(x,y\right)=Det-k\cdot Tr^{2}=\left(AB-C^{2}\right)-k\cdot {\left(A+B\right)}^{2}.$$

This approach was also implemented in SURF (Speeded Up Robust Features) [19], but the keypoints were determined for various scales, and the *k* parameter definition was slightly modified. In the case of KAZE (which means wind in Japanese) [20], the Hessian matrix is found for points at each scale (built using anisotropic diffusion), and a non-maximal suppression suggested in SIFT (Scale Invariant Feature Transform) is also applied. Using a Scharr filter in place of Sobel in the KAZE method is an interesting modification. The STAR approach (also named CenSurE—Center surround extrem es) [21] pays special attention to the necessity of finding precise positions of the keypoints. Therefore, in this latter method, scale-space data are used with the original image resolution without completing subsampling. The Laplacian is computed for all scales and the local extremes are detected. To concentrate on the most characteristic keypoints, the Harris measure and non-maximal suppression over scales are applied. The strength of the keypoint is calculated as a magnitude of the filter response. Moreover, the authors remove keypoints located at the lines by analyzing the eigenvalues of the second moment matrix (equivalent to the Harris measure). It is also worth noting that features that are useful for trackick (GFTT) [22] should have eigenvectors that are larger than a threshold in order to remove the noise effect, and that both should be of similar magnitude.

##### Point Sampling

FAST (Features from Accelerated Segment Test) [6,23], instead of analyzing all pixels of the image patch, focuses on pixels sampled on the circumference of a circle with a radius equal to three pixels, which consists of 16 points. For the rapid version, only four points on the axes are considered, and when three of them are darker or brighter than the central one the corner is found. This comparison requires a threshold, allowing minimal contrast.

AGAST (Adaptive and Generic corner detection based on the Accelerated Segment Test) [7] improves FAST by sampling nine consecutive pixels at the circumference that are brighter or darker than the central point for keypoint location. Next, BRISK (Binary Robust Invariant Scalable Keypoints) [24] calculates FAST-9 for each layer. Then, an approximating function (parabola) is calculated to first find the best scale and then determine the most appropriate coordinates. ORB (Oriented FAST and Rotated BRIEF) [25] also determines the keypoints with FAST-9. However, to find the points of significance, a Harris corner measure is applied.

##### Other Approaches

In SIFT [26,27,28,29], the difference of Gaussian operator is applied to define the pyramid levels. Non-maximal suppression is used to find the extrema. Chosen extrema are then validated by whether they depict a valid corner or by verifying the curvature derived from the Hessian matrix. AKAZE (Accelerated KAZE) [30] detects the keypoints by comparing the gradients of small image patches rather than points themselves. MSER (Maximally Stable Extremal Regions) [31] starts the search for extremal regions by ordering the image pixels according to their intensity. The pixels are then placed in the image and a list of connected components and their areas is calculated. Next, intensity levels that are the local minima of the rate of change in the area function are selected as the threshold for producing MSER.

#### 2.1.2. Keypoint Description

##### Gradient Analysis

**Figure 1:**
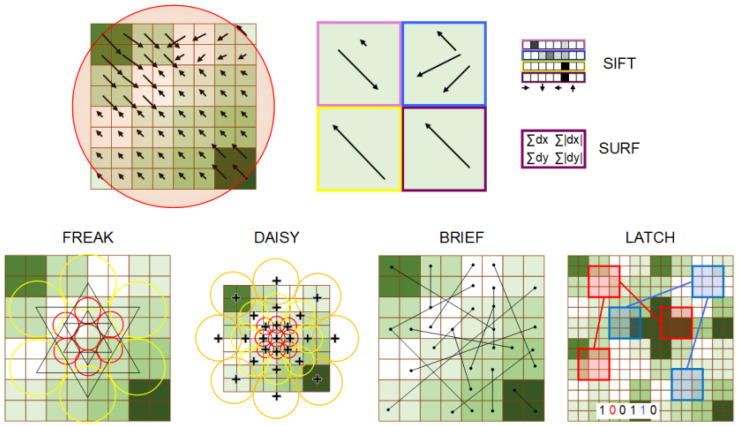
Visualization of the computation of feature descriptors. At the top are examples of features derived from gradients and encoded as real values. At the bottom are different approaches for building a binary string for data description.

SIFT [26,27] aligns the patch according to the orientation defined previously and then the gradients are calculated at a scale of *s* corresponding to the keypoint location. These are additionally weighted with a Gaussian (σ equal to half the width of the descriptor window). The patch is divided into 4×4 regions where the direction histograms of 8 bins are computed. This results in a 128-element feature vector, which is normalized to reduce the impact of lighting variation (see Figure 1 for idea visualization).

The descriptor of the determined keypoint is based on its neighbourhood at a corresponding scale *s*. A distribution of the first-order Haar wavelet responses replaces the gradient calculation when determining SURF [19] features. The KAZE [20] and AKAZE [30] methods follow the above-mentioned approach for keypoint description, but in place of applying Haar wavelet responses to the image transformation these methods use anisotropic diffusion.

##### Point Sampling

BRIEF (Binary Robust Independent Elementary Feature) [32,33] removes noise by applying a Gaussian filter (σ=2) to an image square patch with a size of W=9 pixels. Next, a feature detection function is defined as a test, τ

(4) $$\tau \left(p_{A},p_{B}\right)=\left\{\begin{array}{c} 1\;I\left(p_{A}\right)<I\left(p_{B}\right)\\ 0\;\mathrm{otherwise}, \end{array}\right.$$

where $\left(p_{A},p_{B}\right)$ is a pair of locations defined by a set $n_{d}$ of locations pairs considered to uniquely define the binary test, as depicted in Figure 1. Then, knowing the test responses, a binary string is transformed into a decimal descriptor:

(5) $$E\left(n_{d}\right)=\sum_{1\leq i\leq n_{d}}2^{i-1}\tau \left(p_{A},p_{B}\right),$$

whose resolution depends on the number of tests performed; usually, 128, 256, or 512 comparisons are made.

The creators of the DAISY [34] method for an input image calculate one H orientation map $-+G_{o}$ for each of the quantized directions. Each orientation map is convolved with Gaussian kernels of different values σ, resulting in a daisy-like patch (see Figure 1 for sampling point visualization).

BRISK [24] combines the two approaches described previously; it selects a set $n_{d}$ of interesting points as in BRIEF, and follows the DAISY pattern, as presented schematically in Figure 1. ORB [25] applies rotation normalization, then the BRIEF patch descriptor is used to determine the binary strings describing the patch. FREAK (Fast REtinA Keypoint) [35] reflects the methods used for point selection that have already been mentioned in the previous approaches, but the point sampling corresponds to the receptive field properties of the retina. Another variation is termed LATCH (Learned Arrangements of Three Patch Codes) [36], where instead of pairs of sampled points, triplets of small patches m×m are considered and denoted by the coefficient of the central pixel ($p_{\alpha }$, $p_{\beta }$, $p_{\gamma }$), as presented in Figure 1. The Frobenious norm ($\left|\right|\cdot {\left|\right|}_{F}$) is evaluated to build the comparison, allowing one to create a binary string:

(6) $$\tau \left(p_{\alpha }\right)=\left\{\begin{array}{c} 1\;||p_{\alpha }-p_{\beta }{\left|\right|}_{F}^{2}>||p_{\alpha }-p_{\gamma }{\left|\right|}_{F}^{2}\\ 0\;\mathrm{otherwise} \end{array}\right..$$

As in previous methods, the learning approach for selecting the valid triplets is suggested. This method combines the keypoint descriptors with small texture operators, which are widely represented in different versions of Local Binary Patterns—e.g., CLBP and DTP [37,38].

### 2.2. WrightBroS Database

**Figure 2:**
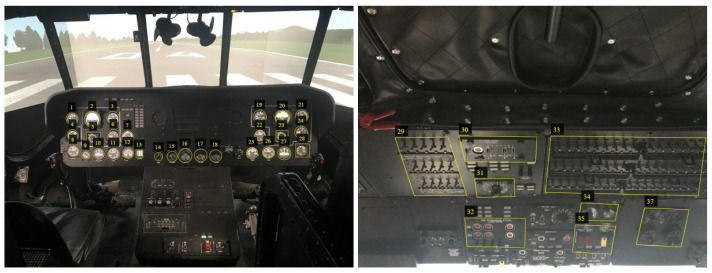
Interior of helicopter cockpit, in which the devices were photographed to prepare the WrightBroS image database. On the left, one can see the front panel. On the right, one can see the top panel.

In the database, we stored images and videos of devices installed in the cockpit of a helicopter flight simulator. Figure 2 depicts the cockpit interior, where the elements of interest on the front panel (on the left) and top panel (on the right) are marked. As shown in the figure, each device selected on the front panel represents a separate class, while on the top panel one class is represented by a set of switches organized next to each other.

**Figure 3:**
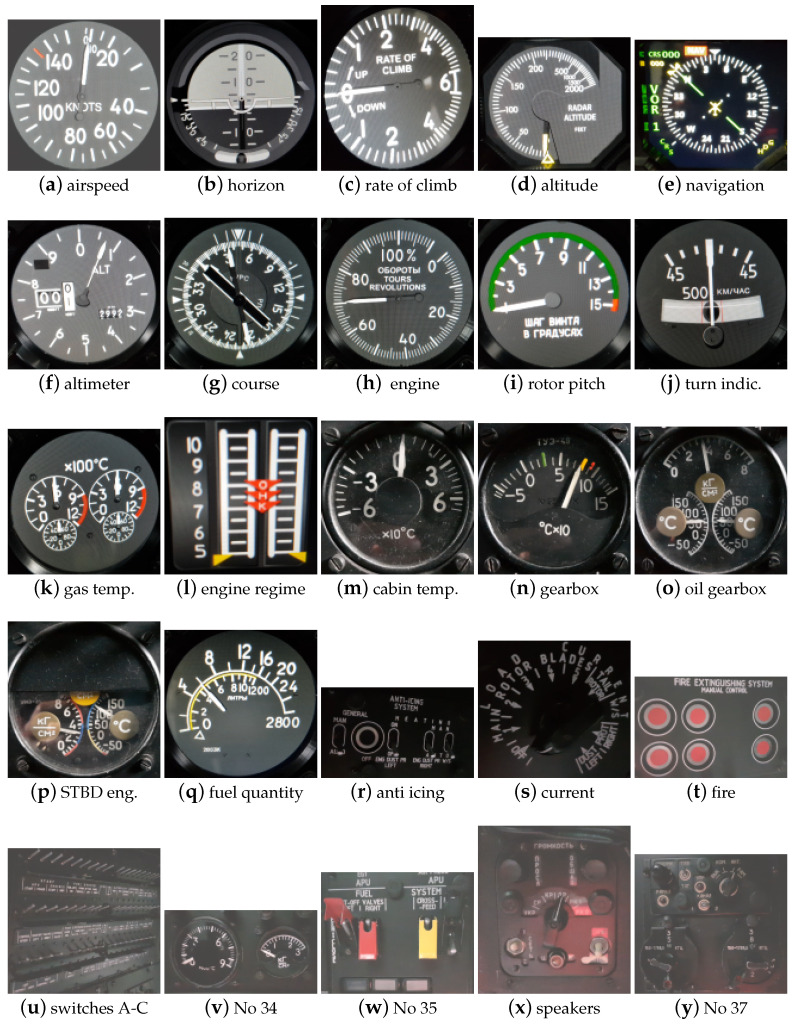
WrightBroS database class representatives.

The database creation process was accelerated by recording videos of selected devices instead of taking a series of photos. The recordings were prepared by different technicians, with various cameras being operated to achieve variable conditions for data acquisition. The technical details of the cameras used are given in Table 1. During each recording, particular attention was paid to reflecting the natural movement of the recording device. Thus, this process was performed manually. Additionally, the technicians were asked to add some reasonable motion. It was assumed that each video would start with the object of interest occupying most of the frame, and that the camera would move away throughout the video, finishing with a general overview of the cockpit. This final assumption simplified the work of the automatic film annotation system, which selected the area of the object from each frame and copied it to the final image database. Data collected by each technician were recorded separately, which allowed us to not only examine the recognition ability of the methods but also to infer the impact of data quality on the results. Figure 3 depicts class representatives, while Table 2 summarizes the number of elements describing each object. The data are accessible online at http://wrightbros.lgnexera.at (accessed on 15 May 2019), the details for access are given in Data Availability Statement.

Videos presenting general cockpit spots complement the database. They show several elements on each frame, making them valuable for use in location precision tests. They were registered by the technicians preparing the database as well as other people, including staff of the Virtual Flight Laboratory at the Silesian University of Technology, thereby giving a greater variation among samples.

### 2.3. Oxford University Database

The Oxford University Database consists of 6 classes of images: bark, bikes, boats, graffiti, trees, and a wall. There are five changes in imaging conditions: viewpoint change, scale change, image blur, JPEG compression, and illumination. Up to two conditions were applied to each image class to assure the separability of the effects from the image content. The image resolution was kept constant within a class but slightly varied within a data set from 765×512 to 1000×700 pixels. All images except the boat class are in color, yet in experiments gray-scale transform is applied. The reasons why this dataset might be chosen are twofold. Firstly, it is the most popular database addressing the problems of feature detection for object recognition and location. Secondly, compared to the WrightBroS database, the limited number of images provided allows for insightful research to be carried out within a reasonable timeframe.

### 2.4. Image Matching

Image matching algorithms need to compare all descriptors computed for an image. This a computationally expensive task due to a large number of descriptors used and their size. The solution searches for short descriptors and a fast comparison function. Therefore, algorithms based on some heuristics to diminish the number of comparisons are preferable. Bearing all these issues in mind, the literature bases the classification on different implementations of k-nearest neighbor (kNN). In all cases, for each keypoint descriptor of an object, the distance and class label of the three closest neighbours must be evaluated. Increasing the distances allows the keypoints to be sorted. Only 15% of the best-matched descriptors are considered in the final rating, neglecting the information contained in vectors characterized by longer distances, as the entire object may not be visible in the image due to it being rotated or overlapping with other objects. Given the label set, the probability of the object belonging to each class is calculated and the class with the highest score is reported.

Many distances could be calculated for multidimensional floating or binary data. The first that comes to mind is the Euclidean distance, which represents the shortest distance between two points. In the presented case, each point is a multidimensional vector given as $\vec{k}=\left(k_{1},k_{2},\ldots ,k_{n}\right)$ and $\vec{m}=\left(m_{1},m_{2},\ldots ,m_{n}\right)$, where *n* is the number of dimensions (in our case, features) and the distance metric is defined as follows:

(7) $$\mathrm{Euclidean}=\sqrt{\sum_{i=1}^{n}{\left(k_{i}-m_{i}\right)}^{2}}.$$

Next, the Mahalanobis distance is the dissimilarity measure between two vectors, $\vec{k}$ and $\vec{m}$, that have the same distribution with a known covariance matrix, *S*:

(8) $$\mathrm{Mahalanobis}=\sqrt{{\left(\vec{k}-\vec{m}\right)}^{T}S^{-1}\left(\vec{k}-\vec{m}\right)}.$$

Both allow for the precise determination of the distance between the data, assuming real values are used. However, for binary data, the time-consuming calculations associated with them generate an excessive overhead, and the use of the Hamming distance, which is calculated for two strings of the same length by comparing characters at the same position, is suggested. The number of character differences corresponds to the distance. In the case of binary data, the use of Hamming distance is preferable due to its rapid means of calculation, even in standard kNN searches. On the contrary, when float data are considered the Mahalanobis or Euclidean distances are considered. Additionally, to improve the timing, the use of the approximated algorithm for kNN is suggested. The FLANN-based (Fast Approximate Nearest Neighbours Search) [39] approach stores the information gathered from the training dataset in several randomized kd-trees and, if necessary, uses the hierarchical k-means tree of the data. The choice of data representation depends on the data themselves and is determined automatically. A randomized kd-tree randomly chooses the split dimension for the first D=5 dimensions on which the data have the greatest variance. A single priority queue is maintained across all the randomized kd-trees. A fixed number of leaf nodes, checked before completing the search and returning the result, determine the degree of approximation. The data points are split using recursive k-means clustering at each level to construct a hierarchical k-means tree. The algorithm stops when K objects are in each cluster. During the search, the algorithm adds to the priority queue all unexplored branches in each node along the path. Then, it extracts the branch that has the closest center to the query point and restarts the search. The approximation degree is defined by the number of iterations of these procedure s.

The method used for the evaluation of the correct classification rate (CCR) in object recognition was as follows. Since the matching procedure assumes a comparison of all descriptors calculated for each image and there are thousands of these, the time taken for the evaluation is considerable. Moreover, in a real-life scenario, this method should work for a small database of training samples. Therefore, the descriptors of ten images were used to train the classifier, while 100 samples were used to test it. However, while the training set may seem small, one should bear in mind that each image is described by hundreds of features. Moreover, according to the experiments we performed (though they are not presented here), the use of more training data does not improve the results obtained.

We performed the classification in two regimes, which both assum e the use of 3-fold cross-validation. In regime A, the data were divided by the technician who made the videos. Since there were three technicians, each dataset prepared by one of them was selected for the testing set (e.g., set I). For training purposes, five images were selected from each of the two other sets (e.g., set II and III). Regime B mixed the data from the entire database, dividing images into three non-overlapping folds with a similar number of samples in the training and testing cohorts (as in previously described case).

## 3. Results and Discussion

Choosing an appropriate method for object detection in images requires the fulfilment of several constraints. In the initial experiments presented in Section 3.1, we focused our efforts on determining which keypoint location method was the most promising one. In these experiments, we investigated the number of keypoints and their location. Next, having chosen the representative number of methods for the keypoint location, the keypoint descriptors were examined. The experiments described in Section 3.2 mainly focused on the time required to carry out the available algorithms. These were executed on a server with an Intel Core i9-7960X with 2.8 GHz CPU and 128 GB RAM. Next, Section 3.3 describes the object recognition performance. The code was written in C++ using OpenCV library version 3.3. In all the experiments, the default settings of the methods were used unless it is stated otherwise.

### 3.1. Keypoints Detection

**Figure 4:**
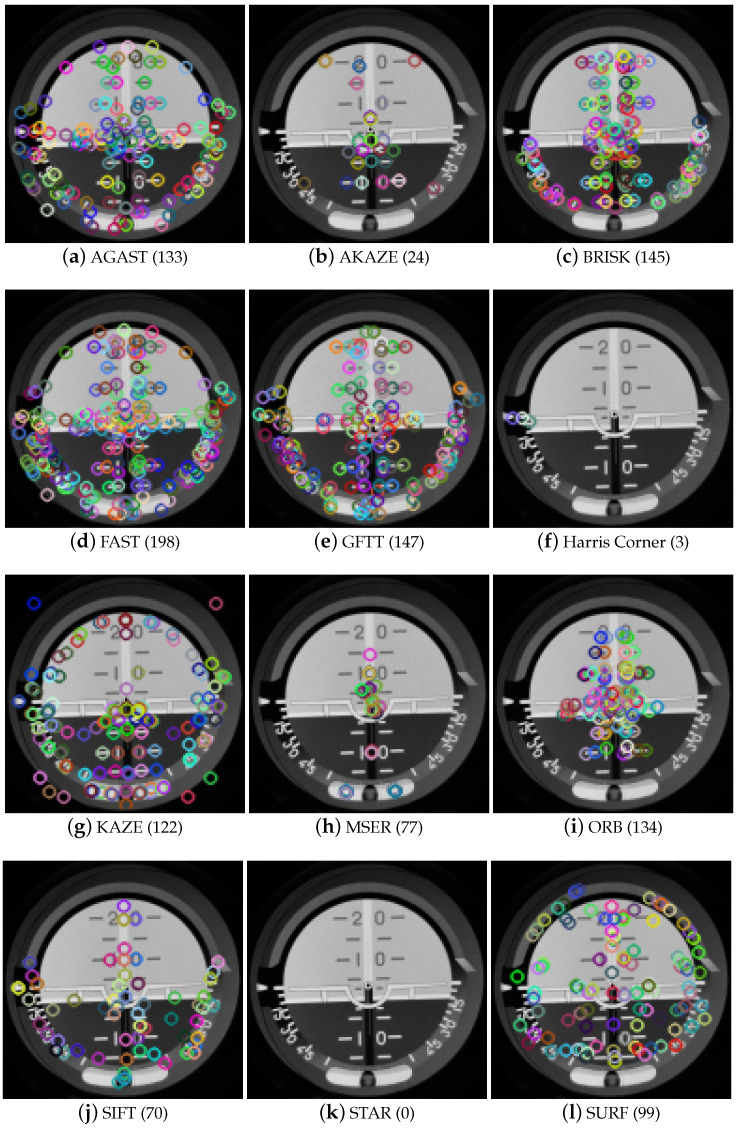
Presentation of the locations of detected characteristic keypoints. The number in the brackets gives the number of locations detected.

We used the Oxford University Database for the verification of the keypoint location quality. Figure 4 presents the characteristic points selected by each of the considered techniques. Generally, the regions selected by keypoints overlap between methods, but details, coordinates, and the number of points var y.

A secondary goal was to verify whether the location of keypoints selected by different methods overlapped. For verification, the keypoints calculated for one randomly selected image were considered. As one can observe in Figure 4, the number of keypoints detected by each method varied. Therefore in Table 3, we present the probability of keypoint repetition between techniques. In the keypoints detected in method A (column) and method B (row), the probability is understood as the ratio of the number of points in set A located in similar positions to those in set B divided by the total number of points in set A. Analyzing these results, when method A has 11 keypoints, method B has 1000 keypoints, and 10 points overlap, one achieves a value of 0.91, but when the order is changed the value becomes 0.01; this is visible, for instance, for GFTT${}_{column}$ – FAST${}_{row}$ and FAST${}_{column}$ – GFTT${}_{row}$. For the measurements considered to be perfect matches (where the distance between the points considered as overlapping was 0) the AGAST and FAST methods showed a high correlation. For an admissible distance of three pixels (presented in Table 3), BRISK and FAST, GFTT and AGAST, GFTT and BRISK, and GFTT and FAST all show a reasonable correlation.

For further processing, the following methods were selected:

- AKAZE as an example of a diffusion method application;
- FAST for its point detection speed and as a representative of AGAST and BRISK;
- GFTT and ORB because they calculate the keypoints in a reasonable time;
- MSER, which uses a different methodology for point selection (despite its performance being too time-consuming, it will be used in further experiments);
- STAR and SIFT for their speed and accuracy, respectively.

**Figure 5:**
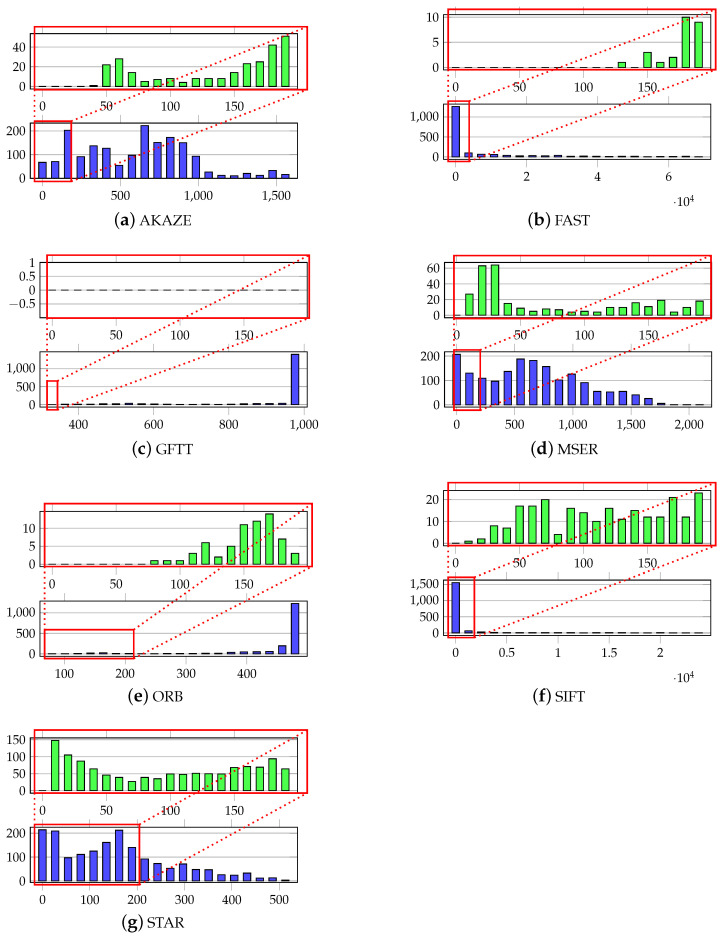
Examples of keypoint distribution over classes in the WrightBroS database. The histogram on the bottom shows distribution over the full keypoint number range, while the one on the top concentrates only on the range [0–200].

Following the conclusion of the keypoint calculation experiments performed with the Oxford University Database, the selected methods were used to prepare keypoint data using the WrightBroS dataset. Since the characteristics of the data differ from those of the first image set, it was necessary to confirm the usability of the chosen methods. We investigated the number distribution of the keypoints within all the images of each class in the WrightBroS dataset. Figure 5 depicts the histograms of the number of keypoints calculated for one class. In each case, it presents the worst scenario, a class in which objects were described as the smallest number of characteristic points. To obtain a good object description, many characteristic points should be found. This is important, because some of these points disappear when object rotation or occlusion take place. The conclusion drawn from our experiments is that a minimum number of 200 keypoints describing an object is sufficient.

An analysis of the data allows one to draw the following conclusions:

**AKAZE:**  The number of keypoints proved to be unsatisfactory. In most classes, the majority of images had less than 200 keypoints. Therefore, we have removed this approach from future experiments. An example of the distribution is presented in Figure 5a.
**FAST:**  The number of keypoints detected in the images had a wide range. Some of the samples only showed several keypoints, but in general the number of selected keypoints was large (around 1000); see Figure 5b.
**GFTT:**  This method detected the maximum number of points, and in the case of this dataset less characteristic points were found only in several cases (see Figure 5c).
**MSER:**  In all the classes, a small number of keypoints describes the largest number of images. However, only the class presented in Figure 5d has images with such a small number of keypoints.
**ORB:**  This method detects the top range of keypoints. Yet, there are some images with a lower number of elements, as depicted in Figure 5e.
**SIFT:**  This method generated the largest amount of keypoints for several images. There were also samples with a low number of characteristic points, but these are comparatively rare; see the example in Figure 5f.
**STAR:**  Similarly to AKAZE, the number of keypoints proved to be unsatisfactory. In most of the cases, the number of keypoints was less than 200; see Figure 5g for example.

### 3.2. Computation Time

**Figure 6:**
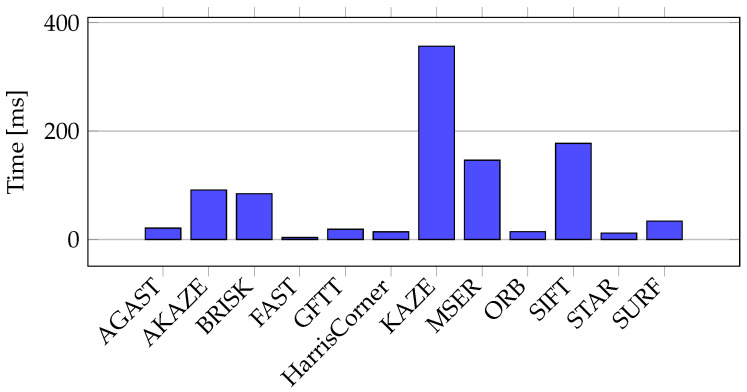
Average time taken for keypoint detection in all images from the Oxford University Database.

The Oxford University Database was also used to perform experiments addressing the time necessary to obtain keypoint locations. Figure 6 shows the significant differences in time performance. The bar plot presents the average time needed to compute one keypoint considering all the points detected by each method. Since the number of detected keypoints per image is relatively large, the influence of system interruptions can be neglected when considering the whole database. Considering the required time efficiency, the execution times recorded in our experiments excluded the use of the KAZE, SIFT, MSER, AKAZE, and BRISK algorithms as keypoint detection algorithms.

**Figure 7:**
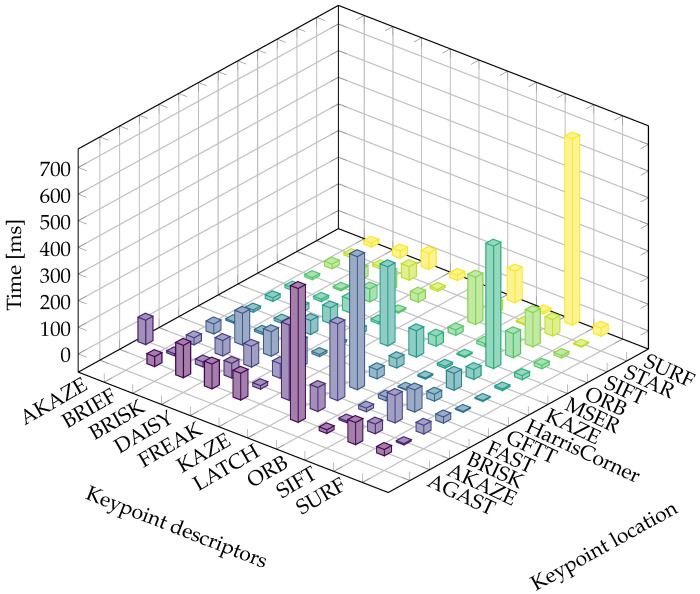
Time taken to calculate the descriptors for all keypoints in the Oxford University Database.

**Figure 8:**
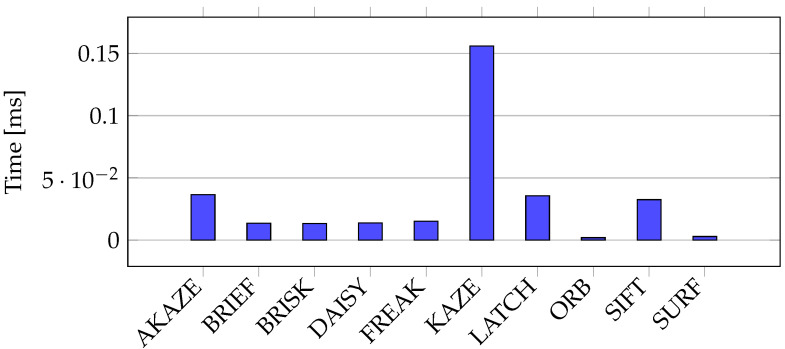
Average time taken to calculate a descriptor for a key point in all images in the Oxford University Database.

Next, the performance of the feature description computation was evaluated using the Oxford University Database. Figure 7 summarizes the total average time taken by each descriptor calculation method, distinguishing between the keypoint location techniques. We observed the computational load resulting from each keypoint description technique when analyzing the bar plot using color. For instance, when AGAST was used for keypoint location, KAZE took the longest time to calculate the descriptions. Conversely, when the SURF method was used to locate the keypoints, SIFT required the longest time period to prepare the descriptors. This plot also depicts the changes in performance when the keypoints were located with the same approach but various methods were used to calculate the descriptor. The average times taken to calculate the descriptions of one keypoint are depicted in Figure 8. As can be seen, the worst performance was found for KAZE. A much better speed was recorded for AKAZE, LATCH, and SIFT. However, a time of 0.04 ms was required for the calculation of a descriptor, which is still too long, as one might be required to calculate thousands of descriptors for each image. ORB achieved the best performance of 0.002 ms, and this was closely followed by SURF, which had a processing time of 0.003 ms. Next were BRIEF, BRISK, DAISY, and FREAK, which could calculate the descriptor in 0.02 ms.

### 3.3. Object Recognition

All these experiments used the image-matching techniques described in Section 2.4. In the case of the binary descriptors (BRIEF, BRISK, FREAK, LATCH, and ORB), the Hamming distance was applied, while for the floating point feature vectors a FLANN-based approach was used. In the second case (DAISY, SIFT, SURF), the experiments with other distance metrics (e.g., L1 and L2) were performed for the selected methods. We omit the detailed presentation of these methods because the outcomes were shown to be similar CCR.

Table 4 shows the classification results achieved by combinations of the methods considered. Along with the average CCR, the standard deviation is presented. The outcomes show that using the FAST and GFTT approaches for keypoint selection results in a weak performance. Neither of the considered techniques provides a good recognition quality for feature description. Yet, in the case of MSER, ORB, and SIFT, several combinations provide promising results.

The BRISK feature descriptor applied to the keypoint prepared using the ORB approach achieved the best score (90.45%). Slightly worse outcomes: 85.35% and 82.59%, were obtained for the FREAK and SURF methods, respectively, when applied for the same keypoints (i.e., keypoints computed by ORB). The best performance for keypoints calculated by the MSER method was achieved when BRISK and SIFT were used to calculate the descriptors, but in this case the CCR did not exceed 78%. Finally, the CCR results for the standard SIFT method fell below 76%.

Table 5 shows the information obtained regarding the correct classification ratio for regime B. We restricted the number of experiments to those combinations which achieved a CCR of at least 50% in regime A. Preparing a database with varying image quality improved the performance in all the considered cases, and the CCR reached a level of 96% for the ORB BRISK combination. Additionally, the significant reduction in the fold variance demonstrated the stability of this approach. To be sure that the results were significantly different, a statistical analysis was performed. We used a t-test to evaluate the null hypothesis that the distribution was similar to a statistical significance level equal to 0.05. This hypothesis did not hold in almost all cases when considering results within groups (columns). The exceptions to this were MESR BRISK andMSER SIFT as well as ORB SIFT and ORB SURF. Next, comparing the best scores from each group, it was found that the hypothesis did not hold up, with a *p*-value equal to 0.0006 being achieved for ORB BRISK and MSER SIFT and a *p*-value of 0.0002 achieved for ORB BRISK and SIFT SIFT.

Bearing these two sets of experiments in mind, it can be concluded that it is beneficial to use data collected by different camera operators and different types of equipment. However, even when this condition is not fulfilled, the proposed solution still allows for very satisfactory object recognition.

## 4. Conclusions

The purpose of these studies was twofold. On the one hand, we aimed to find a quick method for recognizing objects in a flight simulator cockpit that would be useful in an augmented reality system designed in the WrightBroS project. On the other hand, we wanted to explore the possibility of the use of existing methods for locating and describing keypoints.

Firstly, we prepared the WrightBroS database for research purposes. We verified the keypoint location methods that allowed us to choose the most reliable and fast approaches to be FAST, STAR, Harris Corner, ORB, GFTT, and AGAST and determined that AKAZE, BRISK, KAZE, MSER, and SIFT are far slower. We also compared the repeatability of the keypoint location prepared by each of these methods. We concluded that these methods return significantly different locations.

Secondly, we concentrated on the problem of time constraints in keypoint descriptor calculation. The ORB keypoint descriptor was found to be the fastest method. SURF was found to be slightly slower. We recorded far worse performances for the BRIEF, BRISK, DAISY, and FREAK. AKAZE, LATCH, and SIFT methods, which were the slowest to find solutions.

Finally, we evaluated the classification of cockpit elements of the WrightBroS database. Our research showed that methods based on the location of FAST, GFTT and ORB key points have great potential. The best result (90%) was obtained by the use of the ORB keypoint location algorithm followed by the BRISK keypoint descriptor method. We also concluded that when preparing a training set, it is advisable to fill it with images of varying quality. In our experiments, we managed to improve the correct classification ratio to 96%.

## Figures and Tables

**Table 1:** The parameters of the acquisition equipment used for the collection of the WrightBroS database.

Parameter	Camera Set I	Camera Set II	Camera Set III
Smartphone	Samsung Galaxy Note 8	Iphone 7	Samgung Galaxy J3
Lens	f/1.7|f/2.4	f/1.8	f/1.9
Sensor	9.1 MP	12 MP	13 MP
Resolution	1440×1440	4096×2160 (4K)	1920×1080 (fullHD)
Frame rate	30	30	30

**Table 2:** Number of elements in classes of the WrightBroS dataset.

	Set I	Set II	Set III	All
airspeed	1463	2011	1208	4682
altimeter	1762	1486	2089	5337
altitude	1531	1212	2359	5102
antiIcing	819	532	811	2162
course	1249	705	1193	3147
current	1249	431	700	2380
engine	3866	2611	3935	10,412
engineReg	381	238	1018	1637
fire	1075	465	439	1979
fuelQuant	660	682	743	2085
gasTemp	968	502	1505	2975
horizon	1981	1661	2792	6434
mainGear	1057	708	917	2682
mainRotorPitch	715	440	900	2055
navigation	2044	1379	3139	6562
No_34	560	387	516	1463
No_35	569	459	738	1766
No_37	453	102	186	741
oil	790	483	1408	2681
portSTBDEngine	1924	1310	2132	5366
rateOfClimb	1516	1339	1765	4620
speekers	327	23	50	400
switchesA	1051	377	845	2273
switchesB	482	271	287	1040
switchesC	302	287	249	838
tempPass	805	384	846	2035
turnIndicator	2054	1089	2050	5193
Total	31,653	21,574	34,820	88,047

**Table 3:** The percentage of keypoint repetitions (with an up to 3-pixel distance between points considered as overlapping) detected using the considered techniques.

	AGAST	AKAZE	BRISK	FAST	GFTT	HarrisCorner	KAZE	MSER	ORB	SIFT	STAR	SURF
AGAST		0.10	0.76	1.00	0.97	0.17	0.14	0.00	0.86	0.41	0.10	0.14
AKAZE	0.11		0.50	0.21	0.54	0.04	0.86	0.18	0.79	0.61	0.43	0.68
BRISK	0.71	0.40		0.77	0.83	0.17	0.40	0.00	0.94	0.46	0.23	0.40
FAST	0.67	0.15	0.56		0.97	0.15	0.15	0.03	0.85	0.44	0.18	0.10
GFTT	0.06	0.04	0.06	0.10		0.01	0.05	0.03	0.50	0.28	0.04	0.07
HarrisCorner	1.00	0.33	1.00	1.00	1.00		0.33	0.00	1.00	0.67	0.33	0.33
KAZE	0.06	0.44	0.26	0.10	0.34	0.01		0.12	0.28	0.56	0.19	0.65
MSER	0.00	0.10	0.00	0.04	0.24	0.00	0.11		0.14	0.15	0.07	0.19
ORB	0.14	0.11	0.16	0.21	0.87	0.04	0.08	0.04		0.38	0.10	0.12
SIFT	0.07	0.07	0.08	0.10	0.60	0.02	0.18	0.04	0.47		0.06	0.18
STAR	0.14	0.38	0.33	0.29	0.57	0.05	0.38	0.10	0.71	0.52		0.38
SURF	0.05	0.29	0.18	0.05	0.43	0.03	0.57	0.20	0.43	0.54	0.14	

**Table 4:** Regime A: Evaluation of the method’s discriminative capabilities.

Key Point	FAST	GFTT	MSER	ORB	SIFT
Detector	CCR + STD	CCR + STD	CCR + STD	CCR + STD	CCR + STD
BRIEF	29.16 ± 7.75	33.73 ± 8.91	27.39 ± 4.20	37.42 ± 10.85	30.91 ± 7.55
BRISK	14.20 ± 5.97	19.49 ± 8.56	**78.03 ± 6.39**	**90.45 ± 5.05**	32.25 ± 13.94
DAISY	19.43 ± 4.22	25.83 ± 3.00	21.68 ± 4.81	26.36 ± 6.41	25.69 ± 4.24
FREAK	24.70 ± 6.77	27.01 ± 11.38	**65.60 ± 5.01**	**85.35 ± 4.70**	**62.07 ± 9.72**
LATCH	33.35 ± 5.95	35.85 ± 7.70	28.15 ± 4.21	48.60 ± 14.28	42.25 ± 10.49
ORB	18.51 ± 5.27	25.44 ± 7.20	18.40 ± 3.86	**72.94 ± 13.38**	—
SIFT	39.37 ± 10.01	24.27 ± 7.00	**78.72 ± 8.69**	**78.01 ± 6.38**	**76.85 ± 4.15**
SURF	10.10 ± 3.09	8.21 ± 2.49	**50.49 ± 8.68**	**82.59 ± 10.34**	10.63 ± 0.95

**Table 5:** Regime B: Evaluation of the methods’ discriminative capabilities.

Key Point	MSER	ORB	SIFT
Detector	CCR + STD	CCR + STD	CCR + STD
BRISK	86.97 ± 1.68	**95.98 ± 0.76**	—
FREAK	91.68 ± 0.75	92.74 ± 0.94	75.33 ± 0.55
ORB	—	84.99 ± 1.06	—
SIFT	86.42 ± 0.44	90.60 ± 0.96	83.37 ± 0.58
SURF	66.68 ± 2.21	89.28 ± 0.69	—

## Data Availability

The experiments used the publicly available Oxford University Database http://www.robots.ox.ac.uk/~vgg/research/affine/ (accessed on 15 May 2019). Additionally, during the research a WrightBroS database was prepared, which can be accessed online at http://wrightbros.lgnexera.at (accessed on 15 May 2019) with the login reader and password Faziy&Ocuno851.
